# Lessons from lymphatic filariasis elimination and the challenges of post-elimination surveillance in China

**DOI:** 10.1186/s40249-019-0578-9

**Published:** 2019-08-07

**Authors:** Yuan Fang, Yi Zhang

**Affiliations:** National Institute of Parasitic Diseases, Chinese Center for Disease Control and Prevention, Chinese Center for Tropical Diseases Research, WHO Collaborating Centre for Tropical Diseases, National Center for International Research on Tropical Diseases, Ministry of Science and Technology, Key Laboratory of Parasite and Vector Biology, Ministry of Health, 207 Ruijin Er Road, Shanghai, 200025 People’s Republic of China

**Keywords:** *Brugia malayi*, Diethylcarbamazine, Global Programme of Lymphatic Filariasis, Transmission assessment survey, *Wuchereria bancrofti*

## Abstract

**Background:**

The Global Programme to Eliminate Lymphatic Filariasis (GPELF) was launched in response to the call proposed at the 50th World Health Assembly. The goal of the GPELF is to ensure that all the countries where the disease is endemic would have been transmission-free or would have entered post-intervention mass drug administration (MDA) surveillance by 2020. However, several countries are still not on track to discontinue MDA as planned. Thus, issues remain regarding the achievement of stated goals and how to effectively monitor the disease in the post-control and post-elimination phases.

**Main text:**

China was once a lymphatic filariasis (LF) endemic country with heavy disease burden. There were three milestones in the LF control phase of China, including: the proposal that the major focus of the control strategy should be on infectious sources; the three regimens of diethylcarbamazine (DEC) administration according to LF endemic extent; and the establishment of the threshold for LF transmission interruption. It has been ten years since China entered the post-elimination stage (declaration of LF elimination in China was in 2007). Two schemes and a diagnostic criterion were issued to guide all levels of disease control and prevention workers that conduct LF surveillance, as well as those caring for chronic filariasis patients. Regular training courses are held to maintain LF control skills in grass-root institutions. The Notifiable Diseases Reporting System, which included LF in 2004, plays an important role in LF post-elimination surveillance. Until now, no resurgence of LF cases has been detected, except for LF residue foci being found in Fuchuan County of the Guangxi Zhuang Autonomous Region. To confirm that transmission is no longer achievable after a decade since the declaration of LF elimination in China, it is expected within the next two years a transmission assessment survey, conducted in previous LF-endemic areas.

**Conclusions:**

DEC-fortified salt can help accelerate the progress of GPELF before the sprite phase. Sophisticated diagnostic criteria, systematic surveillance regimes, the Direct Network Report system, and regular trainings can effectively prevent the recrudescence of LF during surveillance phases.

**Electronic supplementary material:**

The online version of this article (10.1186/s40249-019-0578-9) contains supplementary material, which is available to authorized users.

## Multilingual abstracts

Please see Additional file 1 for translations of the abstract into the five official working languages of the United Nations.

## Background

### Life-cycle and prevalence of lymphatic filariasis

Lymphatic filariasis (LF) is a mosquito-borne neglected tropical disease (NTD) that is caused by infection from three species of parasitic worms: *Wuchereria bancrofti*, *Brugia malayi*, and *B. timori*. LF is transmitted by five genera of mosquitoes (*Culex*, *Anopheles*, *Aedes*, *Mansonia*, and *Ochlerotatus*) [1]. When microfilariae circulating in the blood stream of an infected human are ingested by a mosquito, they develop into the highly active and infective third larval stage (L3) in 10–12 days. The infective larvae migrate to the mosquito’s proboscis, where they can infect another human host while taking blood meal and, during this process, L3 larvae enter the skin and migrate into the lymphatics and develop into adult worms over a period of 6–12 months [2]. Fertile adult female worms, during their 4–6 year lifespan, release millions of microfilariae into the blood [3].

While LF is rarely fatal, it can cause severe symptoms - lymphedema, elephantiasis, and hydrocele - which induce significant pain and lead to wide-scale loss of productivity and social exclusion [4]. Historically, LF has been endemic in Africa, Asia, the Pacific, and the Americas [5]. Approximately 1 billion people from 72 countries were at-risk for LF infection, with at least 36 million people being affected by the associated morbidity before the World Health Organization (WHO) initiated the Global Programme to Eliminate Lymphatic Filariasis (GPELF) [6].

### Overview of the GPELF and current progress

#### The GPELF proposal

In 1993, based on advances in the diagnosis and treatment of LF, the International Task Force for Disease Eradication classified LF, along with dracunculiasis, poliomyelitis, mumps, rubella, and cysticercosis, as all having the potential for eradication [7]. In 1997, the World Health Assembly (WHA) adopted Resolution WHA 50.29, which called on Member States to initiate activities for the elimination of LF as a public health problem [8]; the GPELF was launched in 2000 as a response to this call [9]. The core objective of GPELF is to eliminate the disease by 2020. Its aims are the interruption of LF transmission with mass drug administration (MDA) in entire populations at-risk, as well as managing morbidities and the prevention of LF-associated disabilities with a minimum package of care [1].

#### Scientific basis of GPELF

Many factors are required for the elimination of LF to become a realistic goal. First, inefficiency in the transmission of LF under the following scenarios: (i) microfilariae can develop, but do not multiply in the vector body; hence, the number of infective larvae is limited by the number of microfilariae ingested; (ii) the infective larvae are deposited on the skin and have to find their way into the bite wound (e.g., approximately 15 500 infective bites of the *Cx. pipiens quinquefasciatus* vector are required to produce a new infectious resource [10], rather than being injected with the mosquito saliva, like malaria sporozoites); (iii) microfilariae can only reproduce when both male and female worms parasitize in the same part of the human body; (iv) mosquitoes die during the development of microfilariae into infective larvae, as they cannot play a role in the transmission cycle [1, 3, 11]. These biological and entomological features show that the transmission efficiency of LF parasites is considered to be less than that of other mosquito-borne parasites, such as *Plasmodium* species. Second, diethylcarbamazine (DEC) or ivermectin (IVM) plus albendazole (ALB) can safely and quickly reduce the prevalence and density of infected individuals, thereby interrupting the source of transmission. Third, *W. bancrofti*, which causes 90% of LF-related morbidities [3], only infect humans, without known animal reservoirs [12]. Furthermore, LF has been eliminated or basic-eliminated in a few countries before or at the beginning of the GPELF, including Japan, China, Australia, and the USA [3]. Of particular interest is the case of China, since it is a developing country that was one of the most heavily endemic for LF at one point [13].

#### Current global progress on the elimination of LF

From 2000 to 2016, 6.7 billion treatments for LF were delivered to more than 850 million people from 66 countries, which considerably reduced the disease transmission in many regions [14]. The implementation of the GPELF, as part of a comprehensive program of NTD control, is entering the final stages. Among the 72 countries where LF was endemic at the start of the GPELF, 14 countries (Cambodia, The Cook Islands, Egypt, The Maldives, The Marshall Islands, Niue, Palau, Sri Lanka, Thailand, Togo, Tonga, Vanuatu, Vietnam, and Wallis and Futuna) have achieved the elimination of LF as a public health problem; seven additional countries have successfully implemented recommended strategies, completed MDA initiatives, and are under surveillance to demonstrate that LF elimination has been achieved [14]. However, MDA is still required in 52 countries and has not been delivered to Equatorial Guinea, Sao Tome and Principe, and South Sudan at the end of 2016, meaning that MDAs will be required beyond 2020 [15]. This conflicts with the GPELF goal of concluding all MDAs by 2020 and moving to the surveillance phase in 100% of the countries where LF is endemic [1].

To accelerate the progress, newly available diagnostic tools (e.g., Alere® filariasis test strip [FTS] [Alere Inc., Scarborough, USA]) would enable more accurate regional quantifications of LF prevalence, as well as any changes after implementation of GPELF strategies [16]. An analysis showed that over 50% of the data reported in some endemic communities of Africa, including coverage data, were inaccurate [17]. It is estimated that the number of implemental units (IUs) requiring MDA could be significantly lower than originally estimated, especially in the high-burden countries of Africa [18, 19]. In the initial implementation of the GPELF, the MDA strategy declared all eligible people in all endemic areas to be administered a single dose of two medicines together once a year, for at least 5 years. However, a few African and southwestern Asian countries either did not complete the mapping or only recently started MDA [20]. According to the report on the mid-term assessment of microfilaremia reduction in the sentinel sites of 13 countries included in the GPELF, for areas with high microfilaria density levels, the efficiency of implementing three rounds of double-drug treatment was lower than 50%; the microfilaria prevalence is far above the transmission interruption threshold, including several sentinel sites in Burkina Faso and Myanmar [21]. In addition, a transmission assessment survey (TAS) failed and MDA needed to be either continued or restarted in some countries where bancroftian filariasis was endemic, especially those where *Aedes* mosquitoes are the primary vector of *W. bancrofti,* such as American Samoa [15]. To accelerate the progress of LF elimination in these countries, alternative MDA regimes are available for areas without loiasis or onchocerciasis co-endemic status. The combination of three medicines (DEC plus IVM and ALB) was recommended, as it can improve microfilaria clearance and provide long-lasting effects [6]. In addition, semi-annual MDA can accelerate the interruption of LF transmission by reducing the number of annual rounds of MDA required to achieve the LF elimination target [20]. Model predictions showed that the semi-annual MDA is likely to shorten the time in half, as well as lower the cost required for LF elimination in countries where it can be implemented [22]. Transmission control can also be achieved through the reduction of human-mosquito contact using vector control approaches, such as sleeping under insecticidal nets and/or indoor residual spraying [23]. LF transmission can be interrupted through the reduction of human-vector contact or microfilariae reservoirs, and by also ensuring that transmission has truly been disrupted and maintaining vector control to prevent recrudescence. Furthermore, effective water, sanitation, and hygiene campaigns, as well as house construction, contributed significantly to the elimination of LF [3, 20]. The past 10 years has led to new treatment regimens, strategies, and diagnostic tools, which have dramatically changed the prospect of LF elimination and would have allowed to the achievement of the GPELF in 2020 for many countries [20, 24].

In the next one and a half years, more countries would have gradually entered the post-control and post-elimination surveillance phases of the GPELF. China is leading the way in LF control and has gathered experiences on these two phases. This paper aims to review and summarize the course of LF control in China, as well as the country’s LF prevention strategy in the post-elimination phase, in order to provide experiences both in the different phases of LF control and in the assessment of LF recrudescence after the validation of transmission interruption.

## Main body

A systematic literature search for relevant articles with the search items of “lymphatic filariasis” and “China” was conducted using the following databases: PubMed, Institute for Scientific Information Web of Science, and China National Knowledge Infrastructure. Only articles fulfilling the following criteria were used for this study: (i) written in English or Chinese; (ii) pertained to evaluation strategies for nation-wide LF control and surveillance; (iii) pertained to the history of LF elimination in China; or (iv) was concerned with the establishment of the threshold for LF transmission interruption and various factors of vector capacity on LF transmission. Additional articles were selected by screening the references of papers that met our inclusion criteria. The following exclusion criteria were applied to titles, abstracts, and full texts: (i) not relating to bancroftian or malayan filariasis; (ii) not relating to target disease control and surveillance; (iii) being a case report or book; and (iv) limited in a local area.

A final set of 15 articles met our inclusion criteria after full-text screening (Additional file 2); five were published in English (33%) and ten in Chinese (67%). Moreover, 11 articles were reviews (73%) on LF control and surveillance strategies, and the remaining four (27%) were research papers. Among these four papers, three focused on the cut-off threshold and one on the vector capacity of bancroftian filariasis transmission.

### Progression of LF elimination in China

#### LF endemic status before control and the four LF elimination phases of progress in China

LF is an ancient disease that was once a major parasitic disease in China. Filariasis due to *W. bancrofti* and *B. malayi* infections was prevalent in China before the initiation of the control programme. A total number of 31 million LF cases was estimated, of which 22 million were bancroftian filariasis and 9 million were malayan filariasis, with 5 million chronic disease manifestations in the 1980s [25]. LF infection involved 16 provinces/autonomous regions/municipalities (P/A/M), which included 864 counties/cities. Of these, 463 counties/cities had bancroftian filariasis, 217 had malayan filariasis, and 184 had mixed infections of both [26]. The specific characteristics of LF prevalence in China were: (i) both *W. bancrofti* and *B. malayi* did not have a reservoir host; and, (ii) the transmission season was short due to the geographic characteristics of most endemic areas in temperate or subtropical regions in China [27, 28]. In addition, the positive rate of natural infection by larvae was relatively low in the vectors, with the highest positive rate of infective larvae being 6.8% (1968) in *An. sinensis*, 32.4% (1957–1959) in *An. lesteri*, 16.1% (1957) in *Cx. pipiens pallens*, and 7.5% (1958) in *Cx. pipiens quinquefasciatus* [29]. Furthermore, with the improvement of living standards and sanitation, as well as the mechanization of agriculture, the mosquito biting rate gradually declined towards the end of the last century [30].

**Fig. 1:**
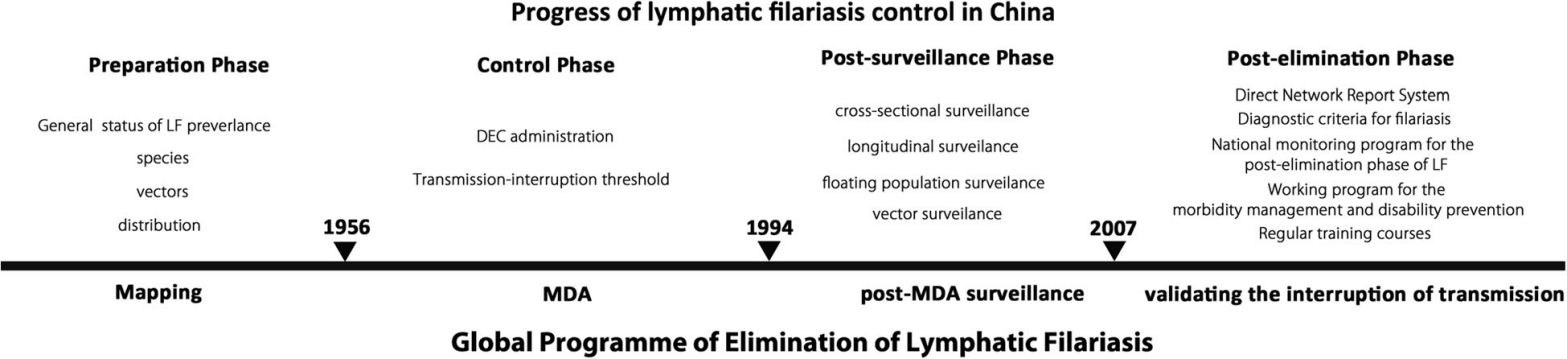
The course of LF elimination in China. The time nodes and important events in the progress of LF control and prevention in China are indicated above each time zone. The corresponding phases of the Global Programme of Lymphatic Filariasis are shown under each time zone. Abbreviations: DEC: Diethylcarbamazine; LF: Lymphatic filariasis; MDA: Mass drug administration

The strategy for LF elimination in China can be divided into four phases - the preparation phase, control phase, surveillance phase, and evaluation of transmission interruption - corresponding to the mapping, MDA and post-MDA phases, and the validation of transmission interruption, according to the GPELF. The core tasks of each phase are shown in Fig. 1. Due to decades of sustained efforts, close cooperation in LF control among government departments, and the active participation of endemic populations, the Chinese Ministry of Health (MOH) officially submitted the national report on the elimination of LF in China to the WHO in March 2006, during the fourth Meeting of the Global Alliance to Eliminate Lymphatic Filariasis, which was held in Fiji [31]. In May 2007, China was officially confirmed as having achieved the elimination of LF as a public health problem by the WHO [29].

#### Contribution of China to the GPELF

The major contribution of China for LF control and the GPELF is the study on the cut-off threshold at which transmission is no longer sustainable [32, 33]. This provides important information for the GPELF to set criteria for evaluating whether MDA has succeeded in lowering the prevalence of infection to a level where recrudescence is unlikely to occur. The three regimes of DEC administration as the principle control strategy of the infectious sources, can safely, widely, and effectively decrease microfilariae rates in a given population (see Table 1). For example, the use of DEC-fortified cooking/table salt, can effectively reduce infectious sources in hyper-endemic areas within 6 months [25, 29]. Based on the biological and entomological features of filariasis and the characteristics of the disease prevalence in China, elimination of infection sources has been established as the major focus of the control strategy [34]. Moreover, the following intervention measures were included in the post-surveillance phase to prevent reemergence of LF: (i) cross-sectional surveillance: based on local LF prevalence, stratified cluster sampling was applied for performing blood examinations in populations from selected administrative villages in each endemic county to monitor the rate and density of microfilaria in multiple cross-sections; (ii) longitudinal surveillance: consecutive data on rate and density of microfilaria were documented in a fixed administrative village in each endemic county every 2 years to observe the dynamics of LF transmission; and (iii) surveillance of floating-populations: blood examinations were performed in populations from areas where LF is endemic and in those from the surveillance area (where the population migrated frequently) for at least 6 months to evaluate the influence of floating-populations on LF control [26, 29, 31]. In addition, mosquito vector surveillance was conducted by detecting the natural infection of filarial larvae in each mosquito, which served as supplemental evidence of the absence of transmission [13]. The economic growth and development, the improvement of utilization of mosquito nets as well as sanitary conditions and health services, have dramatically promoted the progress of LF elimination in China. Most importantly, the success of LF elimination in China is a result of government support, efforts of disease prevention and control workers, and the cooperation of local populations in endemic areas.

**Table 1:** Three regimens for DEC administration applied in different endemic areas of bancroftian and malayan filariasis

Regimen	Endemic extent	Target population	Dose
Selective treatment	hypro-bancroftian filariasis	microfilaraemia positive	3.0 g DEC over 3–5 days 4.2 g DEC over 7 days
	hypro- & meso- malayan filariasis		1.5–2.0 g over 2–3 days
Mass drug administration (usually combined with selective treatment)	hypro- & meso- bancroftian filariasis	> 5 years microfilaraemia-negative	3.0 g over 3–5 days
	meso- & hypro- malayan filariasis		1.0–2.0 g over 2–3 days
DEC-fortified salt	meso- & hypro- bancroftian filariasis	whole population	50 mg DEC/day/person 6 consecutive months (9.0 g in total)
	hypro- malayan filariasis		50 mg DEC/day/person over 3–4 consecutive months (4.5–6.0 g in total)

*DEC* Diethylcarbamazine

### Strategies for post-elimination surveillance of LF in China

As of 2019, China has been confirmed as having eliminated LF (more than 10 years - since in 2007- after the elimination of LF within its borders). China was one of the first few countries to enter the post-elimination phase. Several experiences have been gathered and a few problems were identified during this period. The main objectives of this phase are the surveillance of LF recrudescence and the care of chronic filariasis patients. The Direct Network Report system, strategies for morbidity management and disability prevention concerning chronic filariasis patients, and twice-yearly training courses are the strategies being utilized during post-elimination surveillance.

#### Inclusion of Lymphatic filariasis in the National Diseases Reporting System

Lymphatic filariasis has been included in the Notifiable Diseases Reporting System since 2004. Once a medical institution, including local health clinics in towns and townships, record a piece of information regarding a diagnostic microfilaremia case, the National Institute of Parasitic Diseases (NIPD) will immediately receive an alert message. Subsequently, NIPD officials will inform the provincial center for disease control and prevention to verify the case based on evidence from epidemiology, pathogenic diagnosis, serologic tests, and molecular biologic tests. Annually, approximately 450 direct-network reported cases of LF have been identified over the last 10 years. But, the majority of these cases were false alarms due to misclicking, and the remaining cases were chronic filariasis. Until now, almost no case was identified as microfilaremia through direct-network reporting of the Notifiable Disease Monitoring System, except the Fuchuan case in 2007, which is discussed below.

#### Diagnostic criteria for filariasis

The “Diagnostic criteria for filariasis” was issued by the MOH in 2006. The criteria defined reliable evidence, principle, and criteria for the clinic diagnosis of microfilaremia, as well as acute and chronic filariasis, in different stages of LF (bancroftian and malayan filariasis). The diagnostic of LF should be identified with evidence from epidemiological history (lives in or has travelled to areas where LF is endemic during endemic seasons), clinical manifestations (lymphoedema, hydrocele, chylocele, chyluria), and pathogenic and serological examination. The criteria is applicable to all levels of institutes of disease control and prevention, and medical institutions that diagnosis LF, thereby providing effective means and evidence for LF clinic diagnoses.

#### National monitoring program for the post-elimination phase of LF

In 2010, the “National monitoring program for the post-elimination of lymphatic filariasis” was issued. The surveillance context includes suspected case review, clinical diagnosis, endemic focus disposal, etc. The program was created for the timely discovery of possible residual and imported sources of infection, thereby preventing retransmission of filariasis.

#### Working program for the morbidity management and disability prevention of chronic filariasis patients

When China was confirmed to have eliminated LF, there were approximately 400 000 chronic filariasis patients in previously endemic areas. In 2007, the “Working program for the morbidity management and disability prevention of chronic filariasis patients” was issued by the MOH; the dynamic digital management of chronic filariasis patient documents and the providing of basic personal care for morbidity in local clinical systems were proposed. People with associated chronic manifestations of LF-induced hydrocele, lymphedema, and elephantiasis are treated as chronic LF patients. Care for chronic filarial patients is provided to prevent progression to more advance stages. Notably, improvement in the quality of life of chronic LF patient is a long-term and difficult task in the post-elimination phase. Traditional Chinese and Western medicine approaches were integrated for patients with lymphedema and elephantiasis. Infrared, microwave, or mulberry injection, combined with binding therapy, may help treat chronic filariasis [31]. For patients with scrotal edema, as well as hydrocele and lymphatic scrotum, surgical excision of the diseased skin and scrotal reconstruction can alleviate symptoms. A low-fat, high-protein diet is suggested to patients with chyluria. The clinical severity and progression of the disease, including acute inflammatory episodes, can be reduced and/or prevented by simple measures of hygiene, skin care, exercise, and the elevation of affected limbs. People with lymphedema must have access to continuing care throughout their lives, both to manage the disease and to prevent progression.

In addition, the local health administration should organize the investigation of patients with chronic filariasis to establish a digital disease document for each patient with chronic filariasis for dynamic management. In accordance with the number and distribution of patients with chronic filariasis, related departments should set up care centers in community health service centers or township hospitals (hereafter referred to as community health service institutions), where the patients are relatively concentrated and grass-root protection networks are sound and well-operated; this scenario can provide self-care method guidance and health consultation services for patients with chronic filariasis.

#### National training courses on filariasis prevention and control techniques

National training courses on filariasis prevention and control techniques have been created, with this training being held every 2 years. The attending trainees come from 16 former filariasis endemic provinces. The course covers LF clinical diagnosis, the epidemiology of LF, vector morphology, diagnostics on imported *Onchocerca volvulus* and loiasis, progress on the global elimination of LF, etc. The training helps maintain the ability of individuals to clinically diagnose LF to some extent; however, inadequate in practical skills, especially for the pathogenic detection of filariasis. Since no new LF cases have been detected in over a decade, young medical workers do not have the opportunity to contact with these patients in practice. This is generally a problem in grass-root medical institutions and is a common phenomenon in the field of NTD.

#### Residual foci of bancroftian filariasis found at the beginning of the LF post-elimination phase in China

Although indigenous LF case has not been found in China during the past 10 years, in August 2007, a residual focus of bancroftian filariasis was found in Fuchuan County of the Guangxi Zhuang Autonomous Region. Nineteen cases (1052 persons involved) with microfilaremia were found by pathological examination in Changtang Village with a microfilaria-positive rate of 1.8%; no microfilaria-positive case (4119 residences involved) was found in three other villages in Fuchuan County [35], which attracted the attention of the MOH and WHO. Fuchuan County lies in the region between Guangxi and Hunan provinces. This location was a weak spot during the filariasis control phase, where some microfilariae patients probably did not receive effective treatment or did not take a sufficient amount of DEC-fortified salt. LF was once widespread in China, including distant and remote areas with terrible traffic conditions, especially in the conjunction of these two provinces, which increases the possibility that people received insufficient DEC administration. This means that reservoirs of infection remain in these populations and this scenario may extend the transmission range to the surrounding transmission-interrupting areas as a hidden risk of LF recrudescence. However, this situation also reflects the sensitivity of LF surveillance in post-LF-eliminated China. To further clarify the spread of the remaining affected areas, as well as to assess its impact on the surrounding area, a survey on microfilaraemia in the surrounding region of Fuchuan County and historical filariasis endemic provinces (mainly in Central and Southern China), was conducted between late 2007 and early 2008, using serologic and pathological approaches. No microfilaraemia was found in large-scale filariasis surveillance. Since further investigation revealed that the occurrence of microfilariae cases in Fuchuan County was from residual endemic foci, this was not considered a real resurgence of LF.

#### Transmission assessment survey after a decade since the declaration of LF elimination in China

**Fig. 2:**
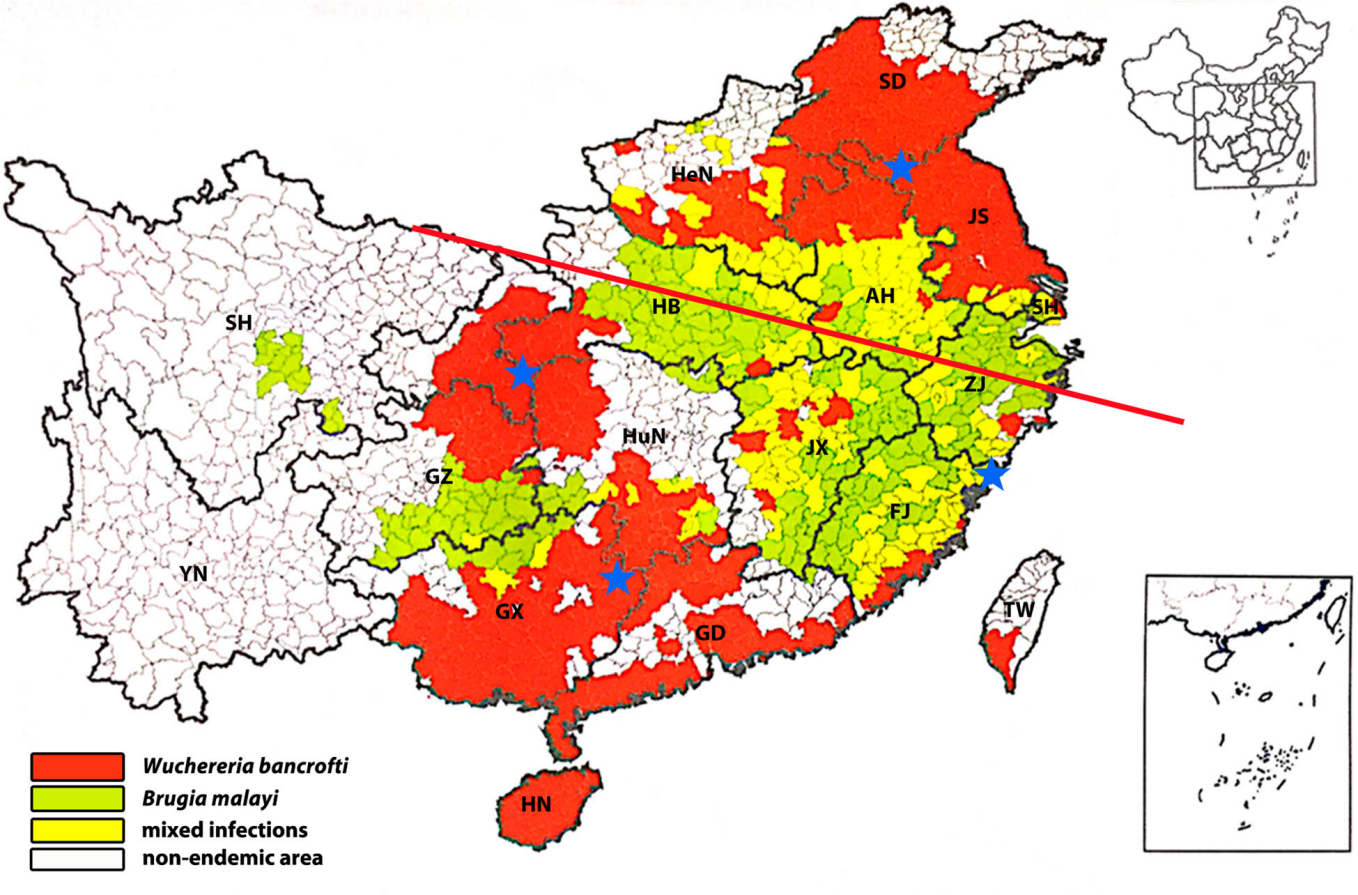
Two evaluation units for transmission assessment surveys of bancroftian filariasis after a decade since the declaration of lymphatic filariasis elimination in China. One is the Middle-Eastern region, and the other is the Central and Southern regions of previous endemic areas. The distribution of endemic areas of lymphatic filariasis in China before control was cited from a previous study [31]. Abbreviations: SD: Shandong Province; JS: Jiangsu Province; ZJ: Zhejiang Province; FJ: Fujian Province; GD: Guangdong Province; GX: Guangxi Zhuang Autonomous Region; HB: Hubei Province; HuN: Hunan Province; GZ: Guizhou Province; SH: Sichuan Province; HN: Hainan Province; YN: Yunnan Province

Although much of the surveillance work was conducted in different periods of LF control in China, residual foci of bancroftian filariasis, such as the incident in Fuchuan County, cannot be completely excluded. Therefore, approximately 10 years after the detection of local filariasis foci in China, a serological surveillance is being expected in four regions with previous bancroftian filariasis epidemics. These efforts are meant to determine if the level of infection is being sustained below the critical threshold of infection under the recent LF surveillance system, as well as to reveal whether recrudescence has occurred in this decade among target population (the design of the survey is provided in Fig. 2). The project is designed to following the TAS, which is conducted to decide whether MDA can be discontinued after multiple rounds of administration [1].

**Fig. 3:**
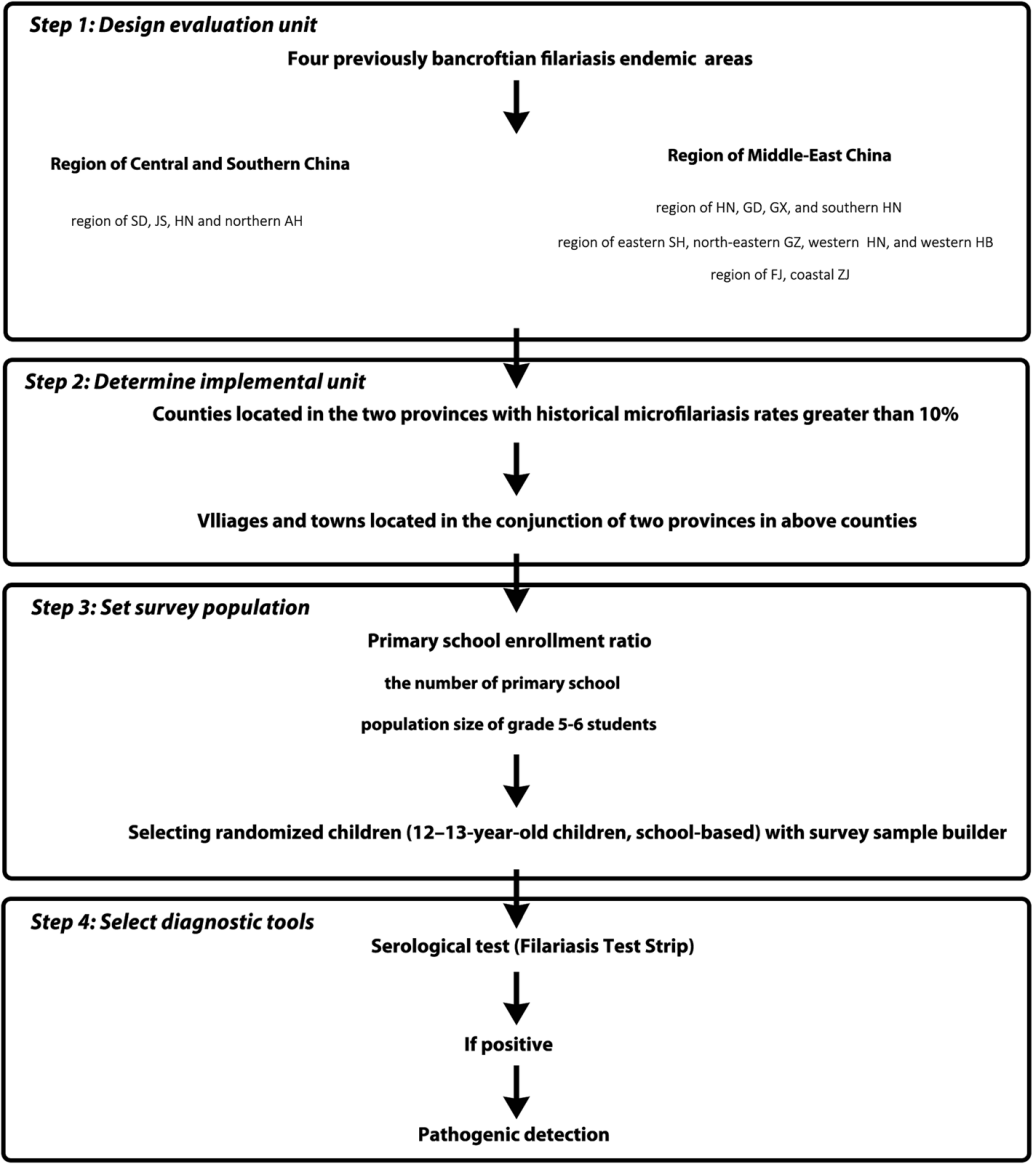
The strategy for transmission assessment survey for bancroftian filariasis after a decade since the declaration of lymphatic filariasis elimination in China. Abbreviations: SD: Shandong Province; JS: Jiangsu Province; ZJ: Zhejiang Province; FJ: Fujian Province; GD: Guangdong Province; GX: Guangxi Zhuang Autonomous Region; HB: Hubei Province; HuN: Hunan Province; GZ: Guizhou Province; SH: Sichuan Province; HN: Hainan Province

The vector of malayan filariasis, *An. lesteri*, was seldom recorded in general mosquito monitoring during this period, although *An. sinensis* is widespread in most areas of China; however, the total population number has dramatically decreased compared with that in the 1980s. In addition, the natural filariasis infectious rate of *An. sinensis* is relatively low as it prefers to blood of livestock [29]. Most importantly, malayan filariasis occupied less than 1/3 of the LF morbidity before the initiation of the control program in China [25]. *Cx. pipiens pallens* and *Cx. pipiens quinquefasciatus*, as vectors of malayan filariasis, are primary mosquitoes in most areas of China that have habitats surrounding humans. Therefore, previous areas where bancroftian filariasis was endemic were chosen as target areas, which do not include malayan filariasis. Since LF has been eliminated in China for over ten years, and no indigenous case been found during this period, the evaluation unit was enlarged to encompass two large-scale, previous bancroftian filariasis-endemic regions (see Fig. 3); one is the Middle-Eastern region and the other is the Central and Southern region. This is different from the general setting of the evaluation unit (EU), which have no more than two million people; although it has the risk that if the critical threshold is exceeded, all IUs that comprise the EU would have to restart DEC administration [1]. Administrative counties located in the conjunction of two provinces with historical microfilariasis rates of more than 10% in each region, were selected as IUs for conducting the TAS.

Children aged 12–13 years, who were born after the declaration of LF elimination in China, were selected as the eligible population. Antigenemia in eligible children is a sensitive marker for relative events of LF transmission for a 12-year period. People who were infected with LF, even those treated with anti-filarial medicines, would retain antigens in the blood for several months or years as the adult worms and microfilariae die and disintegrate [36]. Therefore, detection of the antigen may still be positive, despite a significant reduction in microfilaraemia levels. If the results of LF elimination assessment are consolidated and sustainable, these children should be protected from LF infection. Then, information on the population size of students in grades 5–6 was collected, using the actual primary school enrollment ratio, and the number of primary schools in each IU. Based on the above information, children were randomly selected using the Excel add-in survey sample builder software package [37]. The software is available from the “Neglected tropical diseases support center” (https://www.ntdsupport.org/resources/transmission-assessment-survey-sample-builder) and uses a sampling method based on lot quality assurance sampling [1]. Since the implementation of the 9-year compulsory education initiative in China (24 years ago), the net primary-school enrollment ratio meets the requirement for school-based surveys, which is required for an enrollment ratio greater than or equal to 75%, even for distant and remote villages. This is possible since transportation has been relatively convenient in most of China over the last 15 years due to massive construction and development. Therefore, cluster calculations (school-based) were chosen rather than systematic (enumeration-based) sample calculations. The parameters were selected as follows: *Anopheles* and *Culex* as the principal vectors; and the target threshold was set to be < 2% of antigenemia prevalence. All child participants in the field study provided informed consent; assent by the child and consent from at least one parent were required for children to participate in the study.

In the expected study, FTS for bancroftian filariasis adult worms or microfilariae will be administered to all selected individuals to measure levels of antigenemia. FTSs require no laboratory equipment and can be processed quickly. Moreover, this test has sensitivity, specificity, and stability that are better than the BinaxNow® Filariasis card test (Alere Inc., Scarborough, USA), which has been used by the GPELF for more than 10 years [16, 38]. If the FTS test is positive, program managers can choose to do follow-up testing for microfilaraemia. Peripheral blood is taken at night during the hours of peak microfilariae circulation (between 9 PM and 12 AM) for 3 days continuously, with two blood smears prepared for each person. Each smear contains 60 μl of blood and is examined for microfilaria with a microscope after hemolysis and staining. If LF recrudescence is confirmed by epidemiology and clinical diagnosis, a detail investigation of the extent and range of prevalence will be conducted accordingly. This surveillance project is expected to start in the very near future to evaluate whether or not there is LF recrudescence in China.

#### Limitations of evaluating China for the post-elimination phase of LF

Although LF was eliminated from China in 2007, a number of chronic patients still exist due to past LF infections. In addition, mosquito vectors are still active in previously endemic areas, showing the possibility of local second infections for imported cases. In addition, other than a few microfilaremia cases that were discovered after the 1990s, the practical diagnostic experience for healthcare workers is nonexistent. Moreover, senior experts on LF control have retired and young parasitic diseases control and prevention workers lack experience in the diagnosis and treatment of microfilaraemia cases. Some imported cases of loiasis and *On. volvulus* have been reported in recent years, making filariasis diagnosis more complex [39–41]. Since LF has been eliminated in China, the financial support for LF tasks has been decreasing and several programs for surveillance and morbidity management and disability prevention of chronic filariasis patients cannot be conducted effectively. In addition, compared with other disease control and prevention initiatives, LF is an older field of study; thus, this negatively influences the enthusiasm of young disease control and prevention workers and other medical staffs devoted to LF surveillance.

## Conclusions

Based on the Chinese experience, the administration of DEC-fortified salt in areas where LF is hyper-endemic may effectively reduce the disease prevalence below the threshold of transmission interruption in the final sprite phase of the GPELF. After the entire discontinuation of MDA treatment from 2020 onward, LF surveillance will remain the most important task. In China, in order to achieve the goal of filariasis elimination, the cross-sectional, longitudinal, and floating-population surveillance, supplied with mosquito filariasis carrying surveillance, have been implemented in the post-control phase to survey whether the level of infection is sustaining below the critical threshold of infection. In the post-elimination phase, a diagnostic criterion, and two regimes on LF surveillance and chronic patient care were issued. Furthermore, regular training courses on LF have been held for maintaining the LF diagnostic skills using grass-roots medical training. Finally, the transmission assessment survey is expected to be conducted in the very near future to confirm that transmission is no longer sustainable.

## Additional file

Additional file 1: Multilingual abstracts in the five official working languages of the United Nations. (PDF 477 kb)

Additional file 2: List of articles related to LF control and surveillance in China (citation times were recorded from the China National Knowledge Infrastructure on June 6, 2019). (XLSX 14 kb)

## Data Availability

All data generated or analyzed during this study are included in the published article.

## Abbreviations

ALB: Albendazole
DEC: Diethylcarbamazine
EU: Evaluation unit
FTS: Filariasis Test Strip
GPELF: Global Programme of Elimination of Lymphatic Filariasis
IU: Implemental unit
IVM: Ivermectin
LF: Lymphatic filariasis
MDA: Mass drug administration
MOH: Ministry of Health
NTD: Neglected tropical disease
TAS: Transmission assessment survey
WHA: World Health Assembly
WHO: World Health Organization
