# Supplementary material for: Lessons from lymphatic filariasis elimination and the challenges of post-elimination surveillance in China
Source: Infect Dis Poverty. 2019 Aug 7;8:66. doi: 10.1186/s40249-019-0578-9 (PMC6685173; doi:10.1186/s40249-019-0578-9)
Supplement: Supplementary file 1 — Multilingual abstracts in the five official working languages of the United Nations. (PDF 477 kb) [file 40249_2019_578_MOESM1_ESM.pdf]

Translation of the abstract into the five official working languages of the United Nations

الدروس المستفادة أثناء القضاء على داء الفيل وتحديات مراقبة ما بعد القضاء عليه في الصين

يوان فانغ ويي زانغ

الملخص
معلومات عامة: تم إطلاق البرنامج العالمي للقضاء على داء الفيل (GPELF) وذلك استجابةً للنداء المقترح من جمعية الصحة العالمية الخمسين. الهدف من البرنامج العالمي للقضاء على داء الفيل هو التأكد من أن جميع البلدان التي يستوطن فيها المرض ستكون خالية من انتقال العدوى أو مراقبة تأثير الدواء بعد تواجده في الأسواق بحلول عام 2020. ومع ذلك، ما تزال بلدان كثيرة لا تسير على الطريق الصحيح لوقف MDA كما هو مخطط لها. وبالتالي، لا تزال هناك مشكلات تتعلق بتحقيق الأهداف المعلنة وكيفية مراقبة المرض بشكل فعال في مرحلتي ما بعد السيطرة وما بعد القضاء.
الجسم الرئيسي: كانت الصين ذات يوم دولة يتواجد فيها داء الفيل (LF) المستوطن، والذي كان يشكل عبء كبير. مرحلة التحكم تتكون من ثلاثة مراحل رئيسية لمرض داء الفيل في الصين، بما في ذلك: اقتراح أن يكون التركيز الرئيسي لاستراتيجية التحكم على المصادر المعدية ؛ الأنظمة الثلاثة لثنائي إيثيل كاربامازين (DEC) وفقًا لمدى انتشار مرض داء الفيل؛ وإنشاء حاجز يمنع انتشار داء الفيل. لقد مرت 10 سنوات منذ أن دخلت الصين مرحلة ما بعد الإقصاء (كان إعلان القضاء على داء الفيل في الصين في عام 2007). تم إصدار مخططين ومعيار تشخيصي لتوجيه جميع مستويات العاملين في مجال مكافحة الأمراض والوقاية التي تجري لمراقبة داء الفيل، وكذلك أولئك الذين يرعون مرضى داء الفيل المزمن. خامساً - لأجل رفع قدرات المعلمين في مراكز محو الأمية تعقد لهم دورات تدريبية مستمرة. يلعب نظام الإبلاغ عن الأمراض، والذي شمل داء الفيل في عام 2004، دورًا مهمًا في مراقبة داء الفيل بعد السيطرة عليه. حتى الآن، لم يتم الكشف عن عودة ظهور حالات داء الفيل، باستثناء بؤر يتواجد فيها مرض داء الفيل التي تم العثور عليها في مقاطعة (Fuchuan) بمنطقة (Guangxi Zhuang). للتأكد من أن انتقال العدوى لم يعد ممكنًا بعد عقد من الزمان منذ إعلان القضاء على داء الفيل في الصين، من المتوقع أن يتم خلال العامين المقبلين إجراء مسح لتقييم انتقال، تم إجراؤه في المناطق السابقة المستطونة بالمرض.
الاستنتاجات: يمكن أن يساعد الملح المدعم بـ (DEC) في تسريع تقدم (GPELF) قبل تدهور حالة المريض. يمكن للمعايير التشخيصية المتطورة وأنظمة المراقبة المنهجية ونظام تقرير الشبكة المباشر والتدريبات المنتظمة أن تمنع بشكل فعال استرجاع داء الفيل خلال مراحل المراقبة.

Translated from English version into Arabic by Dina Mohamed Ali, Revised by Amal Alaboud, through

TRANSLATORS
WITHOUT BORDERS

消除淋巴丝虫病以及消除后监测阶段的中国经验

Yuan Fang, De-Jian Sun and Yi Zhang

引言：应第 50 届世界卫生大会决议启动的全球消除淋巴丝虫病规划，旨在于 2020 年在所有丝虫病流行的国家达到丝虫病传播阻断或进入全民服药干预后的监测阶段。然而，目前仍有一些国家未能按计划完成全民服药。因此，如何在 2020 年实现全球消除淋巴丝虫病的既定目标，以及如何在控制和消除阶段有效监测淋巴丝虫病等问题仍然存在。
正文：中国曾是淋巴丝虫病流行最为严重的国家之一，在阻断淋巴丝虫病传播上有 3 个具有

里程碑意义的策略，即明确了以消灭传染源为主导的防治策略，制订了使用乙胺嗪开展群体防治的 3 种方案，确立了淋巴丝虫病阻断传播阈值。自 2007 年世卫组织宣布我国消除淋巴丝虫病已逾 10 年。在此期间，先后颁布了 2 套丝虫病监测方案，1 项诊断标准，用于指导各级疾控和医疗机构开展丝虫病消除后的疫情监测和慢性丝虫病患者关怀照料工作。采用定期举办培训班的形式以在基层医疗机构维持丝虫病临床诊断能力。自 2004 年淋巴丝虫病被纳入全国传染病直报系统以来，在丝虫病消除后监测阶段发挥了重要作用。迄今，未发现淋巴丝虫病在我国复燃，仅在广西省富川县发现一处丝虫病残存疫点。为明确考量我国在实现消除淋巴丝虫病后十余年的监测工作成效，将于未来两年在原丝虫病流行区开展传播阻断评估。
结论：全球消除淋巴丝虫病规划收官在即，乙胺嗪药盐方案有助于加速全球消除淋巴丝虫病的进程。在丝虫病消除后监测阶段，完善的诊断标准、系统的监测方案、高效的网络直报系统系统，以及定期的检测技术培训能有效地防止淋巴丝虫病的再流行。

Translated from English version into Chinese by Yuan Fang

Filariose lymphatique : leçons à tirer concernant son élimination et enjeux liés à la surveillance après la phase d'élimination en Chine

Yuan Fang et Yi Zhang

Résumé
Contexte : Le Programme mondial pour l'élimination de la filariose lymphatique (GPELF) est né d'une volonté exprimée à l'occasion de la 50e Assemblée mondiale de la santé.
L'objectif du GPELF est de veiller à ce que, d'ici 2020, tous les pays dans lesquels la maladie est endémique soient exempts de transmission ou amorcent la surveillance post-traitement médicamenteux de masse (TMM). Toutefois, plusieurs pays sont loin d'être prêts à mettre un terme à la TMM dans les délais prévus. Il reste donc à lever certains obstacles pour que les objectifs fixés soient pleinement réalisés et parvenir à assurer une surveillance efficace de la maladie dans les phases de post-contrôle et de post-élimination.
Corps du texte : La Chine a autrefois été un pays endémique de la filariose lymphatique (FL), avec pour caractéristique une lourde charge de morbidité. La phase de contrôle de la FL y reposait sur trois axes : (i) la proposition plaçant les sources infectieuses au premier plan de la stratégie de contrôle ; (ii) l'administration des trois doses de diéthylcarbamazine (DEC) selon l'étendue de la zone endémique de la FL ; (iii) la définition d'un seuil d'interruption de transmission de la FL. La Chine entrait en phase post-élimination il y a dix ans (la déclaration d'élimination de la FL en Chine a eu lieu en 2007). Toutes les équipes responsables de la surveillance et de la prévention de la FL, ainsi que les fournisseurs de soins aux patients atteints de filariose chronique, ont pu s'appuyer sur la mise en place de deux programmes et d'un critère diagnostique. De la formation est organisée régulièrement dans les institutions locales afin que le personnel préserve ses acquis en matière de surveillance de la FL. Le Dispositif de signalement des maladies à déclaration obligatoire, auquel la FL a été ajoutée en 2004, joue un rôle primordial dans la surveillance lors de la phase post-élimination. Jusqu'à présent, aucun cas de FL n'a été rapporté, exception faite de foyers résiduels de FL dans le district de Fuchuan, dans la région autonome Zhuang du Guangxi. Afin de confirmer

l'absence de risque de transmission dix ans après l'annonce de l'élimination de la FL en Chine, une enquête d'évaluation de la transmission (TAS) sera menée dans les deux années à venir dans d'anciennes zones d'endémie de la FL.
Conclusion : Le sel enrichi en DEC peut contribuer aux objectifs du GPELF en amont de la phase d'éclosion. Divers facteurs peuvent prévenir la recrudescence de FL pendant les phases de surveillance, notamment le recours à des critères diagnostiques élaborés, la mise en place de systèmes de surveillance systématique, le système de Référence directe du réseau, ainsi que la formation continue des intervenants.

Translated from English version into French by Erling Prevost, Revised by Eric Cote, through

TRANSLATORS
WITHOUT BORDERS

Уроки, извлеченные из опыта ликвидации лимфатического филяриоза, и сложности постэлиминационного надзора в Китае

Юань Фан и И Чзан

Аннотация
Введение: Глобальная программа по ликвидации лимфатического филяриоза (The Global Programme to Eliminate Lymphatic Filariasis, GPELF) создана в рамках инициативы, предложенной на $50^{й}$ Всемирной ассамблее здравоохранения. Цель GPELF - убедиться, что во всех странах, где болезнь эндемична, её носительство остановлено или введен надзор за последствиями массового применения лекарственных препаратов к 2020 году. Однако некоторые страны все еще не перешли в фазу прекращения массового применения лекарственных препаратов, как было запланировано. Таким образом, остаются сложности с достижением поставленных целей и с определением эффективных способов мониторинга заболевания на стадиях, следующих за контролем и элиминацией.
Основная часть: Лимфатический филяриоз (ЛФ) в Китае был широко распространенным эндемичным заболеванием. Фаза контроля над ЛФ в Китае включала три основных этапа: предположение, что главной стратегической целью должен быть контроль источников заражения; три режима лечения с помощью диэтилкарбамазина (ДЭК) в соответствии с распространением эндемичного ЛФ; установление порога прекращения передачи ЛФ. Прошло 10 лет с того момента, когда Китай перешел на стадию постэлиминации (заявление о ликвидации ЛФ в Китае было сделано в 2007). В качестве руководства для работников, которые проводят надзор за ЛФ на всех уровнях контроля и предотвращения заболевания и заботятся о пациентах с хроническим филяриозом, были выпущены две схемы и диагностический критерий. Для поддержания навыков контроля ЛФ в местных учреждениях проводятся регулярные учебные курсы. Система отчетности по инфекционным заболеваниям, подлежащих регистрации, в которую с 2004 года включен ЛФ, играет важную роль в постэлиминационном надзоре за ЛФ. До сих пор не было выявлено ни одного случая

повторного возникновения ЛФ, кроме остаточного очага ЛФ, обнаруженного в округе Фучуань автономного региона Гуанси-Чжуан. Чтобы подтвердить, что спустя десять лет после заявления о ликвидации ЛФ в Китае распространение заболевания остановлено, в течение двух лет будут проведены исследования по оценке передачи в местах,ранее эндемичных для ЛФ.
Заключение: Обогащенная ДЭК соль может помочь ускорить Глобальную программу борьбы с лимфатическим филяриозом до начала инвазивной стадии. Сложные диагностические критерии, системное управление надзором, система прямого оповещения и регулярные учебные курсы могут эффективно предотвращать повторные вспышки ЛФ в течение фазы надзора.

Translated from English version into Russian by Valentina Simonova, Revised by Anna Kukharchuk, through

TRANSLATORS WITHOUT BORDERS

Lecciones de eliminación de filariasis linfática y los retos de vigilancia post-eliminación, en China

Yuan Fang y Yi Zhang

Resumen
Antecedentes: El Programa Mundial para Eliminar la Filariasis Linfática (GPELF; *por sus siglas en inglés*) se puso en marcha, en respuesta al llamamiento propuesto en la 50th Asamblea Mundial de la Salud. El objetivo de la GPELF es garantizar que todos los países, en los que la enfermedad es endémica, hayan estado libres de transmisión o hayan entrado en vigilancia de administración de medicamentos, en masa, (MDA) después de la intervención, para el año 2020. Sin embargo, varios países todavía no están en camino de interrumpir MDA, como estaba previsto. Por lo tanto, sigue habiendo problemas con respecto al logro de los objetivos establecidos y cómo monitorear, efectivamente, la enfermedad, en las fases de post-control y post-eliminación .
Conjunto principal: China fue una vez un país endémico de filariasis linfática (LF) con una gran carga de enfermedad. Hubo tres hitos en la fase de control de la LF, en China, incluyendo: la propuesta de que el enfoque principal de la estrategia de control debería estar en las fuentes infecciosas; los tres regímenes de administración de dietilcarbamazina (DEC), de acuerdo con la extensión endémica del LF; y el establecimiento del umbral para la interrupción de la transmisión LF. Han pasado 10 años, desde que China entrara en la etapa posterior a la eliminación (la declaración de eliminación de LF en China fue en 2007). Se publicaron dos esquemas y un criterio de diagnóstico, para guiar a todos los niveles de los trabajadores de control y prevención de enfermedades, a que llevan a cabo la vigilancia de la LF, así como a los que atienden a pacientes con filariasis crónica. Se llevan a cabo cursos de capacitación regulares, para mantener las habilidades de control de LF, en instituciones de base. El Sistema de Notificación de Enfermedades

Notificables, que incluyó la LF, en 2004, desempeña un papel importante, en la vigilancia posterior a la eliminación de la LF. Hasta ahora, no se ha detectado ningún resurgimiento de los casos de LF, excepto que se encuentran focos de residuos de LF, en el condado de Fuchuan, de la región autónoma de Guangxi Zhuang. Para confirmar que la transmisión ya no se puede lograr, después de una década desde la declaración de eliminación de LF en China, se espera que en los próximos dos años se realice una encuesta de evaluación de transmisión realizada en anteriores áreas endémicas de LF.
Conclusiones: La sal fortificada por DEC puede ayudar a acelerar el progreso de GPELF, antes de la fase de sprites. Los sofisticados criterios de diagnóstico, los regímenes de vigilancia sistemática, el Sistema de informes de Red Directa y la formación regular pueden prevenir, eficazmente, el recrudecimiento de la LF, durante las fases de vigilancia.

Translated from English version into Spanish by María Jose, Revised by María Luz Puerta, through

TRANSLATORS
WITHOUT BORDERS
